# Comparison of Antero-Lateral Thigh Flap and Vastus Lateralis Muscle Flap for the Treatment of Extensive Scalp Defects—A Retrospective Cohort Study

**DOI:** 10.3390/jcm12196208

**Published:** 2023-09-26

**Authors:** Julius Moratin, Philip Dao Trong, Karl Semmelmayer, Jan Mrosek, Sven Zittel, Moritz Bleymehl, Oliver Ristow, Christian Freudlsperger, Jürgen Hoffmann, Michael Engel

**Affiliations:** 1 Department of Oral and Cranio-Maxillofacial Surgery, University of Heidelberg, Im Neuenheimer Feld 400, D-69120 Heidelberg, Germany; karl.semmelmayer@med.uni-heidelberg.de (K.S.); mrosek@mkg-langen.de (J.M.); sven.zittel@med.uni-heidelberg.de (S.Z.); moritz.bleymehl@med.uni-heidelberg.de (M.B.); oliver.ristow@med.uni-heidelberg.de (O.R.); christian.freudlsperger@med.uni-heidelberg.de (C.F.); juergen.hoffmann@med.uni-heidelberg.de (J.H.); michael.engel@med.uni-heidelberg.de (M.E.); 2 Department of Neurosurgery, University of Heidelberg, Im Neuenheimer Feld 400, D-69120 Heidelberg, Germany; philip.daotrong@med.uni-heidelberg.de

**Keywords:** free flap, muscle flap, scalp defect, head and neck, reconstruction

## Abstract

Free flap reconstruction is the standard of care for extensive defects of the head and neck area. In this study, two types of free flaps, the antero-lateral thigh flap (ALT) and the vastus lateralis muscle flap, were compared. The primary endpoint was flap success, secondary endpoints were complication rates, hospitalization and surgery time. Cases with defect situations of the scalp and consecutive microvascular free flap reconstructions using either ALT flaps or vastus lateralis muscle flaps between 2014 and 2022 were retrospectively analyzed. Indications, perioperative handling and outcomes were compared. Twenty patients were included in the analysis. Ten patients (50%) received a free flap reconstruction using an ALT flap and ten patients (50%) received a vastus lateralis flap. A simultaneous two-team approach was possible in each case and the flap success rate was 100% with the need for one successful anastomosis revision. The mean defect size in our cohort was 147 ± 46 cm^2^. There were no significant differences in surgery time, duration of hospitalization or complication rate between both cohorts. Both free flaps, the ALT and the vastus lateralis flap, are suitable for the closure of large scalp defects. They provide high success rates, short surgery times without the need for patient repositioning and low donor-site morbidity. The vastus lateralis muscle flap bares the advantage of being perforator-independent and allows for the preparation of long vessels for anastomosis if needed while baring the disadvantage of a prolonged period of healing via granulation or the need for secondary surgery in terms of covering by split-thickness skin grafts which may interfere with necessary adjuvant treatment in oncological patients.

## 1. Introduction

Scalp defects may follow after ablative tumor surgery, trauma or as a consequence of wound healing disorders secondary to cranial surgery. Depending on their localizations, size and the medical history of the affected patients, they may impose a relevant challenge on the treating specialist. In many cases, defect situations affect soft and hard tissue and lead to the necessity to provide both, hard tissue reconstruction via cranioplasty, and the restoration of soft tissue [1]. While minor defects may be treated with primary wound closure or local flaps, free tissue transfer using microvascular free flaps offers the possibility to adequately treat extensive defects [2,3,4]. Apart from aesthetical considerations, sufficient wound closure in cranial defects is of clinical relevance to prevent the spread of infections, especially in extensive defects with intracranial communication and when cranioplasty was performed [5,6].

Several types of free flaps have been presented for defect restoration of the cranium and each type has distinct features that potentially may influence peri- and postoperative procedures and clinical outcomes [7]. Studies on clinical outcomes in free flap reconstruction in the head and neck area are numerous and several factors with potential influence on flap success have been described, including a history of radiotherapy, vascular diseases and previous surgical procedures [8,9]. Those risk factors often are present in patients after sequential surgical procedures to the cranium due to recurrent tumors or wound healing disorders after decompressive surgery.

The goal of the present study, therefore, was to investigate a cohort of patients with extensive cranial defects who were treated in an interdisciplinary approach including neurosurgeons and reconstructive oral and maxillofacial surgeons. Two different types of free flaps were used, the antero-lateral thigh (ALT) flap and the vastus-lateralis muscle flap, and compared regarding success rates and peri- and postoperative management.

## 2. Materials and Methods

### 2.1. Patients and Data Acquisition

The presented study was designed as a retrospective single-center cohort study to compare two different free flaps for the closure of extensive cranial defects (i.e., antero-lateral thigh flap—ALT and vastus lateralis muscle flap—VL). The primary endpoint was flap success, secondary endpoints were complication rates, hospitalization and surgery time. We retrospectively analyzed the medical records of patients receiving free flap reconstruction of the head and neck area in the Department of Oral and Cranio-Maxillofacial Surgery of the Heidelberg University Hospital between September 2010 and November 2021. All patients receiving a free flap reconstruction to the cranium with or without cranioplasty were included in this analysis.

The study was conducted in accordance with the principles stated in the Declaration of Helsinki. Approval of the local ethics committee was given (ethic vote: S-513/2017) and written informed consent was obtained from all patients.

Clinical data were collected focusing on perioperative data including initial diagnosis and indication for the reconstructive approach, type of flap used in each case, pedicle vessels, duration of surgical procedures and hospitalization, and peri- and postoperative morbidity and complications.

### 2.2. Surgical Procedures

Patients were treated surgically by the Department of Oral and Maxillofacial Surgery with or without the participation of the Department of Neurosurgery in cases where cranioplasty needed to be performed. All patients received wound closure with a free flap (either ALT or vastus lateralis flap). All surgical procedures were performed in a simultaneous two-team approach to economize surgery time. In some patients with cutaneous squamous cell carcinoma elective or therapeutic neck dissection was performed additionally. The recipient vessels were chosen intra-operatively with regard to size and localization of the defect, pedicle length of the flap and the need to perform a neck dissection. No preoperative diagnostics to determine recipient vessels were performed.

### 2.3. Statistical Analysis

Statistics were performed using SPSS Statistics^®^ 25 (IBM, Armonk, NY, USA). Clinical and demographical data were analyzed and summarized with descriptive statistics. Comparison of categorical data was performed with Chi-squared testing and Student’s *t*-test was used for comparison of mean values of unpaired parameters. A *p*-value of 0.05 or less was considered to indicate statistical significance.

## 3. Results

### 3.1. Patient Cohort

An overall number of 20 patients were included in the analysis. Eight patients were female (40%) and 12 were male (60%). The mean age was 65 ± 19 years (range from 27 to 96 years). The reasons for the reconstructive approaches were wound healing disorders following cranial surgery in 10 cases (50%) and cutaneous tumors in 10 cases (50%). Further information on the patients, including medical history is provided in Table 1 and Table 2.

The affected subsites of the cranium included the parietal region in 11 cases (55%), the temporal region in 8 cases (40%), and the occipital region in 1 case (5%).

### 3.2. Surgical Procedures and Perioperative Management

All patients received a free flap for defect closure. Ten defects (50%) were treated with an ALT flap and ten defects (50%) were treated with a vastus lateralis muscle flap. Cranioplasty was performed in 7 patients (35%) using palacos cement in 4 patients (57%), patient-specific titanium implants in two patients (29%) and Polymethyl methacrylate (PMMA) in 1 patient (14%). The treated skin defects ranged from 10 cm to 18 cm in diameter with a mean defect size of 145 ± 47 cm^2^ (range: 80–252 cm^2^).

The facial artery and the internal jugular vein were the vessels mostly used for anastomosis in the cohort. Table 3 provides detailed information on the vessels used for anastomosis. In 1 patient (5%) a venous interposition graft was used to enhance the pedicle length in order to facilitate the anastomosis. Six patients (30%) with cutaneous squamous cell carcinoma received an ipsi- or bilateral neck dissection according to tumor localization and preoperative CT scan within the same surgery.

Primary closure of the donor site defect could be achieved in 18 patients (90%), while secondary healing/closure happened in two patients (10%) who received a cranial defect closure with an ALT flap. Supplementary Figure S1 exemplifies the donor sites of two patients following ALT harvesting with primary closure in one patient and secondary healing via granulation in another patient.

**Figure 1:**
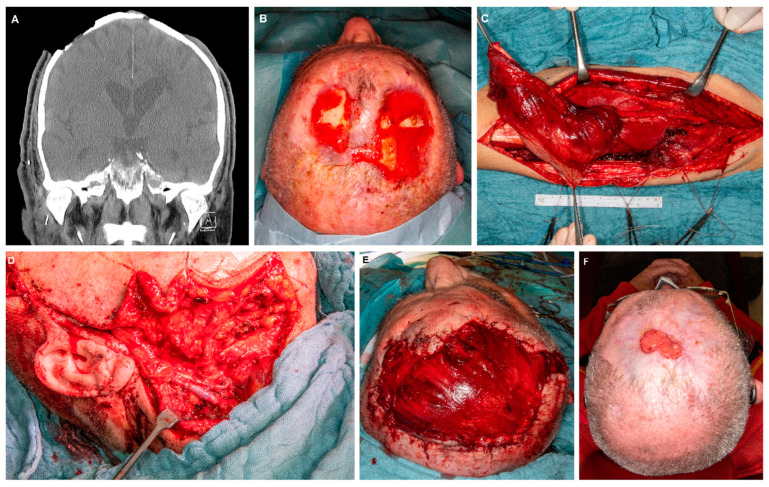
Example of a cranial reconstruction using a vastus lateralis muscle flap in a patient with two cutaneous squamous cell carcinomas of the parietal region. (**A**): CT scan presenting the primary tumor region with infiltration of the calvarial bone. (**B**): Image of the preoperative situation after partial tumor resection. (**C**): Intraoperative situation of the harvesting procedure of the muscle flap. (**D**): Intraoperative situation after anastomosis of the pedicle vessels to the superior thyroid arterial and the facial artery. (**E**): intraoperative situation after soft tissue reconstruction with the vastus lateralis muscle flap. (**F**): situation after 9 months during secondary healing (i.e., conservative therapy via wound coverings until epithelialization).

**Figure 2:**
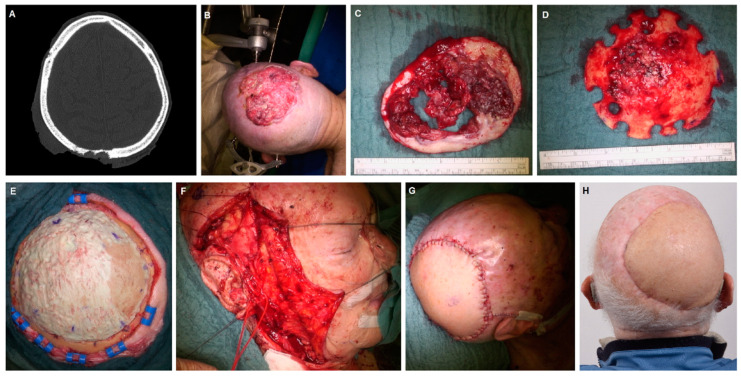
Example of a cranial reconstruction using an ALT flap in a patient with a cutaneous squamous cell carcinoma of the scalp. (**A**): CT scan presenting the primary tumor region with infiltration of the calvarial bone. (**B**): Image of the intraoperative situation before tumor resection. (**C**): Resected soft tissue. (**D**): Resected calvarial bone. (**E**): Intraoperative situation after coverage of the osseous defect with Polymethyl methacrylate (PMMA). (**F**): Image of the intraoperative situation after anastomosis of the pedicle vessels to the facial artery and the facial vein. (**G**): Intraoperative situation after soft tissue reconstruction with the ALT flap. (**H**): Postoperative situation two months after surgery.

**Figure 3:**
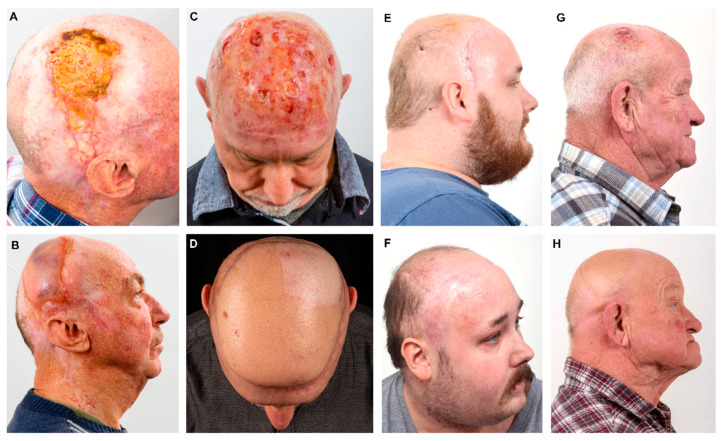
Pre- and postoperative situations in four patients with free flap reconstruction of the scalp. (**A**,**B**): Patient with cutaneous squamous cell carcinoma of the parietal region of the right side; (**A**): preoperative situation, (**B**): postoperative situation (3 months after reconstruction with VL flap—size: 15 × 10 cm)—muscle under granulation. (**C**,**D**): Patient with angiosarcoma of the capillitium; (**C**): preoperative situation, (**D**): postoperative situation after tumor resection and reconstruction with ALT flap (size: 20 × 15 cm). (**E**,**F**): Patient with wound healing disorder after therapy of an astrozytoma; (**E**): preoperative situation with wound healing disorder in the temporal region of the right side, (**F**): postoperative situation 1 year after reconstruction with VL muscle flap (size: 15 × 8 cm) and after full epithelialization. (**G**,**H**): Patient with cutaneous squamous cell carcinoma; (**G**): preoperative situation, (**H**): postoperative situation after tumor resection and reconstruction with ALT (size: 10 × 8 cm).

Table 4 gives an overview of perioperative management, complications and hospitalization in dependence of the used flaps. There were no significant differences regarding all investigated parameters between the two groups. The mean surgery time for the whole cohort was 273 ± 71 min and did not differ between the ALT and the vastus lateralis group (Table 4). The mean hospitalization in the ICU was 8 ± 15 days (median: 2.5 days). The mean hospitalization was 18 ± 13 days (median: 14.5 days). Figure 1 and Figure 2 illustrate the preoperative status, perioperative management and surgical results of cranial defect closures using a vastus lateralis muscle flap (Figure 1) and an ALT flap (Figure 2), respectively. Figure 3 provides pre- and postoperative impressions on four patients treated with vastus lateralis muscle flaps or ALT flaps.

### 3.3. Complications

The flap success rate in this cohort was 100% and revision of the anastomosis was successfully performed in one patient (5%) with an ALT flap. Major complications including postoperative cardio-pulmonary deterioration occurred in four patients (20%) and led to prolongated hospitalization (Table 4). Three patients with major complications suffered from recurrent SCC and one patient from recurrent meningioma with extensive wound healing disorders.

## 4. Discussion

Defect situations of the scalp mostly follow after ablative surgery of cutaneous tumors or as a consequence of wound healing disorders after cranial surgery. Especially composite defects of the skin and the calvarial bone that may result after multiple surgeries or multimodality treatment of cranial pathologies require adequate treatment to prevent secondary complications like intracranial spread of infections. Large defects and those arising after complex pre-treatment, often including multiple surgical procedures with or without radiotherapy, in most cases require the use of free flaps in order to provide sufficient tissue for a reliable reconstruction [10,11]. Several different strategies and algorithms have been described for the treatment of scalp defects and a variety of free flaps have been described as reliable options. Those include the latissimus dorsi free flap for large defects, the parascapular flap, the radial forearm flap and the anterolateral thigh flap (ALT) among others [7,12,13,14,15,16,17]. Other alternatives for extensive defects, like the omentum flap that has been described for pharyngeal reconstruction, bear the necessity for intraabdominal flap harvest, and, thus, are usually not the first choice for scalp reconstruction [18,19].

In the present study, we aimed to present our experience with the treatment of scalp defects using our in-house favorite flaps for this indication, the ALT and the vastus lateralis muscle flap. While the ALT has often been described as the standard of care for large scalp defects among others, as stated above, the vastus lateralis (VL) flap seems to be far less commonly in use. While the VL has been described as a versatile treatment option for soft tissue defects of the head and neck in some studies, up to date, there are hardly any comparative studies on its advantages and disadvantages when used for the treatment of scalp defects [20,21,22,23,24,25].

The ALT flap provides a skin paddle which makes it the first choice for extraoral defect closure. This applies even more in patients suffering from malignant tumors (e.g., cutaneous squamous cell carcinomas) with an urgent necessity for timely adjuvant radiotherapy. Correspondingly, the use of the vastus lateralis flap implies the need for primary or secondary covering of the muscle. This may be regarded as a disadvantage as secondary surgery is time-consuming for the patient and the treating specialist and carries the usual risks of surgical procedures. Furthermore, as stated above, it may delay or even impede the prompt execution of adjuvant therapy. While it may be possible to cover the muscle using a split-thickness skin graft in the primary procedure, in our department the muscle is left uncovered in order to be able to monitor the flap perfusion during the critical postoperative phase. After a period of around 4–8 weeks of granulation, the patient either will receive a secondary covering of the muscle using split-thickness skin grafts, or, depending on the patient’s choice, wound coverings until full epithelialization as a conservative alternative. A further disadvantage of the VL muscle flap is the shrinkage of the muscle during healing. This has been observed by other authors as well and requires a certain over-dimensioning of the muscle to prevent secondary soft tissue retraction with exposure of calvarial bone or materials used for its reconstruction [23,25]. This necessity of over-dimensioning is partly due to the mentioned shrinking of muscle, and partly to the nature and form of the VL muscle itself, which impedes a preparation as a flat wound covering. Although the muscle allows for a certain shaping to adapt it to the corresponding defect, the nutrient vessels necessitate a certain flap thickness, which leads to a bulky appearance during the first 4–8 weeks. In the course of granulation; however, the muscle usually shrinks and integrates into the surrounding skin level. Covering of the muscle with split-thickness skin grafts may usually be performed after 4–8 weeks. Alternatively, if the patient refuses further surgical approaches, a conservative approach with wound coverings until full epithelialization is chosen. The period until full epithelialization was highly variable between the patients and ranged from 3–12 months after surgery. An advantage of the vastus lateralis flap, on the other hand, is its independence of perforator vessels and its long pedicle [24]. Being perforator-independent, it exhibits far fewer anatomical variations of the pedicle vessels than the ALT and may be used as an alternative to the ALT in case no sufficient perforators can be found during surgery or if a long pedicle vessel is needed, e.g., if the temporal vessels are insufficient for anastomosis and the cervical vessels have to be used as a consequence. Considering the disadvantages of muscle flaps mentioned above, the standard flap for cranial defect closure in our department is the ALT. In cases of unreliable perforators or the need for very long pedicle vessels, however, the intraoperative change to harvesting a VL muscle flap, in our experience provides a safe and time economic solution without the need for the establishment of additional donor sites (e.g., ALT of the other leg, latissimus dorsi etc.), and without the risk of a compromise regarding the safety of the anastomosis, and, consequently, the flap success. Another argument in favor of the muscle flap is related to its reported tendency to shrink. While initial overdimensioning is warranted, the flap settles to the level of the surrounding tissue during healing. The ALT, however, in many cases requires thinning of the skin paddle due to its tendency towards increased bulkiness in Western countries [7,26]. An oversized skin paddle, however, may either be accepted with a risk of aesthetical disadvantages and impaired flap perfusion due to increased tissue pressure or may be corrected by time-consuming thinning of the ALT. The thinning of skin paddles is a matter of discussion with several authors describing it as a safe procedure, while other authors reported an elevated risk of damaging the perforator vessels and, thus, of a partial or complete flap loss as a consequence [27,28,29].

The pedicles in both flaps are long and of solid caliber with the perforator vessels being a weak point of the ALT.

The mean defect size in our analysis was 153 ± 50 cm^2^ with a range from 80 to 300 cm^2^. The comparison of both groups revealed no significant differences between the defect sizes of patients treated with an ALT flap and those treated with a VL flap, respectively. Weitz et al. described scalp defect sizes of >1000 cm^2^ in their analysis and advocated the use of latissimus dorsi flaps in those cases. While ALT and VL flaps may be limited to certain defect sizes, we did not find patients with defects exceeding 300 cm^2^ in our records and, thus, successfully treated all patients with cranial defects and the need for free tissue transfer with either ALT or VL flaps [7].

For anastomosis, the superficial temporal vessels have been described as a reliable and safe option for the reconstruction of cranial defects by several authors [7,30]. In our cohort, however, the temporal vessels were only chosen for anastomosis in five patients (25%). This may be explained by weak caliber temporal vessels in some patients, and the necessity to perform a neck dissection in some patients with cutaneous squamous cell carcinoma (n = 6; 30%) that granted access to the cervical vessels anyway. The low rate of revision surgeries and flap losses in our cohort, however, suggests that the intraoperative exploration and evaluation of vessels is a safe procedure and preoperative imaging that has been suggested by other authors seems unnecessary in most cases.

Harvesting ALT flaps may lead to different degrees of donor site morbidity depending on the position of the perforator vessels and the size of the required skin paddle. Harvesting VL flaps, in contrast, necessarily leads to the resection of relevant parts of the muscle with the consequence of permanent functional impairments of this motor unit. There are numerous publications indicating comparably low donor-site morbidity and functional impairment after ALT and vastus lateralis flap harvesting, with postoperative reduction of sensitivity of the upper thigh being the most commonly mentioned side effect. Nevertheless, a more detailed examination of the postoperative muscle function revealed a significant decrease in knee flexion, walking speed, and, health-related quality-of-life in patients after VL flap harvesting [20,31,32,33]. While it probably can be assumed that the functional impairment after VL flap harvesting may be more relevant than after ALT harvesting, the primary closure of the donor site that can be achieved at 100% after VL flap harvesting may be regarded as an argument in favor of muscle flaps as it spares the patient time-consuming wound treatments in cases of secondary healing, and possibly allows for an earlier mobilization. While the use of muscle flaps may be regarded as critical in general due to the mentioned aspects, especially in oncological patients with a need for adjuvant therapy, there is a relevant number of publications advocating its use in scalp reconstruction, mainly under reference to their size and reliability.

Considering the mentioned factors, in our opinion, the ALT flap is the method of choice for cranial defect closure. The VL muscle flap, however, provides a safe and reliable option in cases of weak caliber perforator vessels or the need for long pedicle vessels, especially in patients without the urgent need for timely adjuvant radiotherapy.

## 5. Conclusions

Both flaps, the antero-lateral thigh (ALT) flap, and the vastus lateralis muscle flap were shown to be reliable options for the reconstruction of extensive scalp defects with no relevant differences regarding hospitalization and treatment-related complications. While usage of the vastus lateralis flap leads to the need for a certain over-dimensioning and secondary coverage of the muscle, it presents a safe strategy when no or only insufficient perforator vessels prevent the use of an ALT flap.

## Figures and Tables

**Table 1:** List of included patients with relevant demographic and clinical data (RT: radiotherapy; ALT: antero-lateral thigh flap; VL: vastus laterals muscle flap).

ID	Gender	Age	Reason for Surgery	Prior RT	Used Flap
1	female	66	Wound healing disorder (cerebral aneurysm)	No	ALT
2	male	96	Cutaneous SCC	No	ALT
3	male	27	Wound healing disorder (Glioma)	Yes	VL
4	male	78	Cutaneous SCC	No	VL
5	female	54	Melanoma	No	ALT
6	female	40	Wound healing disorder (cerebral aneurysm)	No	VL
7	male	78	Cutaneous SCC	Yes	ALT
8	female	80	Wound healing disorder (Osteomyelitis)	No	ALT
9	male	95	Cutaneous SCC	No	VL
10	male	78	Cutaneous SCC	No	ALT
11	male	75	Cutaneous pleomorphic sarcoma	No	VL
12	female	62	Wound healing disorder (Osteoradionecrosis)	Yes	VL
13	male	77	Cutaneous SCC	No	ALT
14	female	40	Wound healing disorder (Meningioma)	Yes	VL
15	female	33	Wound healing disorder (Glioma)	Yes	VL
16	female	65	Wound healing disorder (cerebral aneurysm)	No	VL
17	male	66	Meningioma	No	ALT
18	male	71	Wound healing disorder (Osteoradionecrosis)	Yes	VL
19	male	78	Cutaneous SCC	No	ALT
20	male	60	Wound healing disorder (Meningioma)	Yes	ALT

**Table 2:** Information on medical history of the patients including prior treatment, comorbidities and reasons for cranial defects.

Medical History of Patients		*n* (%)
Reasons for Cranial Defects		
Wound healing disorder		10 (50%)
	Cerebral aneurysm/SAH	3 (30%)
	Glioma	2 (20%)
	Osteomyelitis	1 (10%)
	Meningioma	2 (20%)
	Osteoradionecrosis	2 (20%)
Tumors		10 (50%)
	Cutaneous squamous cell carcinoma (SCC)	8 (80%)
	Malignant melanoma	1 (10%)
	Meningioma	1 (10%)
Relevant comorbidities		
	Cardiovascular	8 (40%)
	Metabolic	9 (45%)
Prior treatment		
	1–3 operations	6 (30%)
	>3 operations	14 (70%)
	Prior radiotherapy	7 (35%)

**Table 3:** Vessels used for anastomosis in dependence of flap.

Arteries	ALT Flap	VL Flap	Total
Superficial temporal artery	4 (80%)	1 (20%)	5 (25%)
Facial artery	4 (50%)	4 (50%)	8 (40%)
Superior thyroid artery	2 (40%)	3 (60%)	5 (25%)
Lingual artery	-	2 (100%)	2 (10%)
Veins	ALT Flap	VL Flap	Total
Superficial temporal vein	4 (80%)	1 (20%)	5 (25%)
Facial vein	1 (20%)	4 (80%)	5 (25%)
Internal jugular vein	4 (50%)	4 (50%)	8 (40%)
External jugular vein	1 (50%)	1 (50%)	2 (10%)

**Table 4:** Comparison of categorical data and mean values of treatment in dependence of used free flap (*p*-Values for comparison of categorical data according to Chi-squared test; *p*-Values for comparison of mean values according to Student’s *t*-test).

Parameter		ALT Flap	VL Flap	*p*-Value
Indication for surgery	Tumor	6 (60%)	4 (40%)	0.371
	Wound healing disorder	4 (40%)	6 (60%)	
Closure of donor site	Primary closure	8 (80%)	10 (100%)	0.136
	Secondary closure	2 (20%)	-	
Flap revision	Yes	1 (10%)	-	0.305
	No	9 (90%)	10 (100%)	
Major complications	Yes	2 (20%)	2 (20%)	1.0
	No	8 (80%)	8 (80%)	
Hospitalization, surgery time and defect sizes				*p*-Value
Mean surgery time (minutes)		288 (±81)	258 (±59)	0.351
Hospitalization—ICU (days)		10 (±20)	5 (±7)	0.476
Hospitalization (days)		22 (±17)	14 (±5)	0.180
Mean defect sizes		154 ± 61	152 ± 21	0.962

## Data Availability

The data presented in this study are available on request from the corresponding author.

## Supplementary Materials

The following are available online at https://www.mdpi.com/article/10.3390/jcm12196208/s1, STROBE statement, Supplementary Figure S1. Depiction of donor sites of two patients. (**A**): Left upper thigh after harvesting of ALT flap and primary wound closure. (**B**): Right upper thigh after harvesting of ALT flap during secondary healing of the donor site defect via granulation.
