# Supplementary figures and images for: Comparison of Antero-Lateral Thigh Flap and Vastus Lateralis Muscle Flap for the Treatment of Extensive Scalp Defects—A Retrospective Cohort Study
Source: J Clin Med. 2023 Sep 26;12(19):6208. doi: 10.3390/jcm12196208 (PMC10573281; doi:10.3390/jcm12196208)

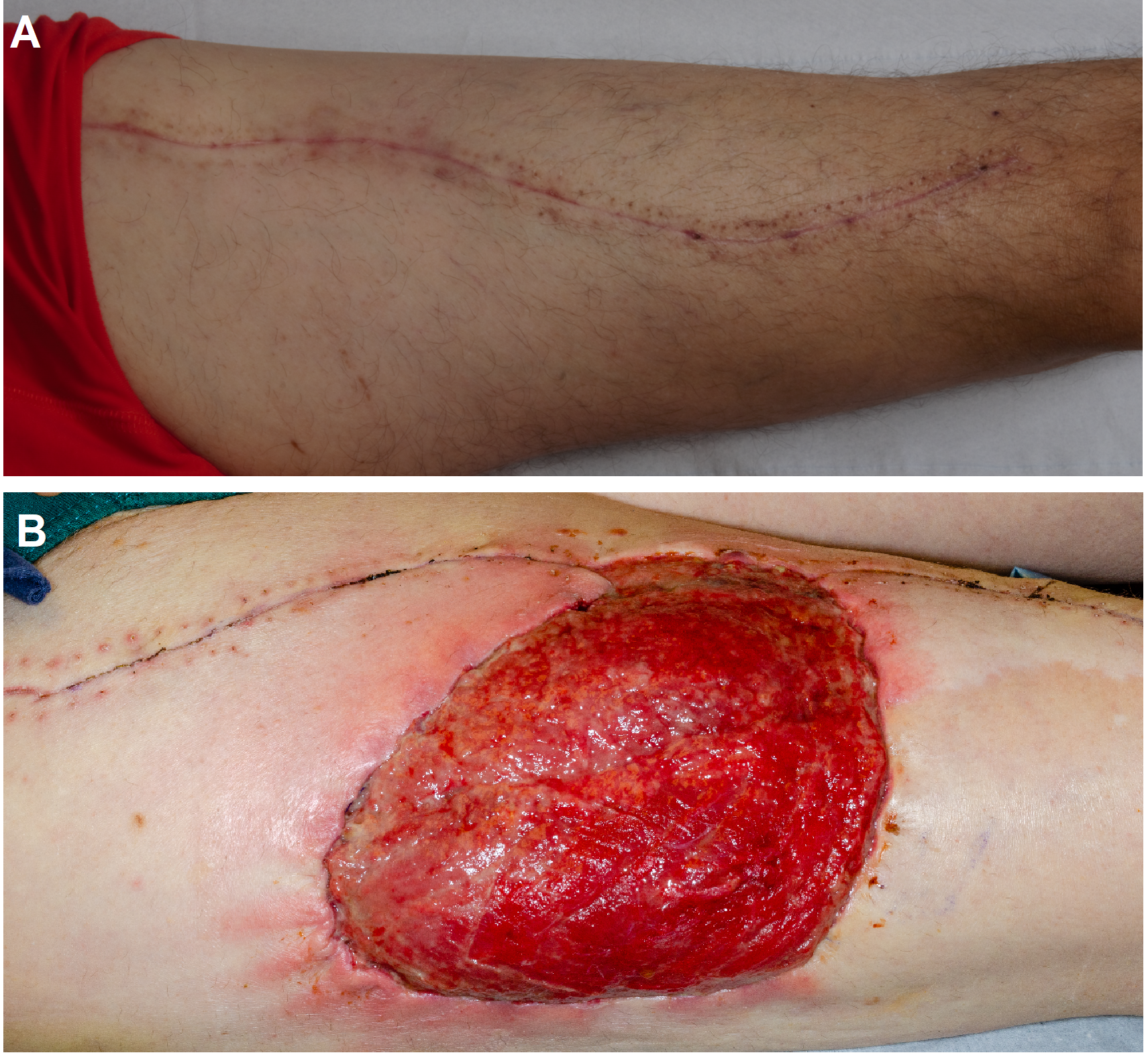

Supplement: Supplementary file 1 [file jcm-12-06208-s001.zip › Supplementary Figure S1.tif]
